# International research priorities for integrated care and cross-boundary working: an electronic Delphi study

**DOI:** 10.1093/intqhc/mzae095

**Published:** 2024-09-27

**Authors:** Jason Scott, Justin Waring, Aaron Asibi Abuosi, Yakubu Adole Agada-Amade, Jibril Muhammad Bashar, Aoife De Brún, Henry Cann, Philip Crowley, Lindsay H Dewa, Samantha Spanos, Siri Wiig

**Affiliations:** Faculty of Health and Life Sciences, Northumbria University, C115, Coach Lane Campus, Newcastle upon Tyne NE7 7XA, United Kingdom; Health Services Management Centre, University of Birmingham, Park House, 40 Edgbaston Park Road, Birmingham B15 2TT, United Kingdom; Department of Health Services Management, University of Ghana Business School, Legon, Accra PO Box LG78, Ghana; Department of Health Administration and Management, Faculty of Health Sciences and Technology, College of Medicine, Enugu Campus, University of Nigeria, Enugu 400241, Nigeria; Department of Standards and Quality Assurance, National Health Insurance Authority, POW Mafemi Crescent, off Solomon Lar Way, Utako District, Abuja 900108, Nigeria; Department of Standards and Quality Assurance, National Health Insurance Authority, POW Mafemi Crescent, off Solomon Lar Way, Utako District, Abuja 900108, Nigeria; UCD Centre for Interdisciplinary Research, Education and Innovation in Health Systems (UCD IRIS Centre), School of Nursing, Midwifery & Health Systems, University College Dublin, Belfield, Dublin D04 V1W8, Ireland; Innovation and Improvement, The Health Foundation—Q, 8 Salisbury Square, London EC4Y 8AP, United Kingdom; The Health Service Executive (HSE) Strategy and Research, HSE, Dr Steevens Hospital, Steevens Lane, Dublin DO8 W2A8, Ireland; School of Public Health, Imperial College London, White City Campus, London, Westminster W12 0BZ, United Kingdom; Australian Institute of Health Innovation, Macquarie University, 75 Talavera Rd, North Ryde, Sydney, New South Wales 2109, Australia; Centre for Resilience in Healthcare (SHARE), Faculty of Health Sciences, University of Stavanger, Stavanger, Rogaland N-4036, Norway

**Keywords:** integrated care, cross-boundary working, Delphi

## Abstract

**Background:**

Integrated care can be broadly defined as the delivery of high-quality and safe care for patients as they cross organizational boundaries or when care is delivered with multiple health care teams, professions, or organizations. Successful integration of care services is contingent on multiple and complex factors across macro, meso, and micro levels of health and social care systems in lower-, middle-, and higher-income countries. Previous priorities for the future development of integrated care have focused on designing and implementing models or approaches to integrated care rather than establishing the research needed to underpin them. This study aimed to address this evidence gap by developing a consensus on international research priorities related to integration of care and cross-boundary working.

**Methods:**

We conducted a sequential electronic Delphi (eDelphi) study from September 2023 to December 2023. The eDelphi process consisted of initial priority generation followed by two rounds of consensus development via an online survey. Sixty-six priorities were generated by 19 delegates at an international conference workshop titled, ‘Priority setting for future research on integration of care and cross-boundary working’. Workshop delegates then identified other experts in integrated care and cross-boundary working from their networks. In each eDelphi round, participants then provided item-by-item responses using a seven-point Likert scale, with consensus defined *a priori* as ≥80% agreement (strongly agree or agree). Priorities that reached consensus were conceptually grouped into topics.

**Results:**

Twenty-five of 66 unique (37.9%) research priorities achieved consensus after two eDelphi rounds. In Round 1, 63/85 (74.1%) experts from 10 countries across 4 continents achieved consensus on 12/66 (18.2%) priorities. In Round 2, 51/63 (81.0%) experts achieved consensus on a further 13/54 (24.1%) priorities. From the 25 priorities, we derived six conceptual groupings that represent broad topics for future research on integrated care and cross-boundary working: (i) access to care, (ii) data sharing and technology, (iii) measurement of care quality, (iv) patient experience and satisfaction, (v) service design, integration and governance, and (vi) teamwork and leadership.

**Conclusion:**

Integrating care services and improving cross-boundary working is important for improving the quality of care provided to patients, regardless of country. Therefore, the conceptual topics and individual priorities identified in this study can inform policymakers, practitioners, and researchers when designing or evaluating integrated care services across the world in pursuit of improved integrated care systems.

## Introduction

Multiple definitions of integrated care exist and are context-specific [1], but integrated care can be broadly defined as the delivery of high-quality and safe care for patients as they cross organizational boundaries or when care is delivered with multiple health care teams, professions, or organizations [2, 3]. Health and social care system contexts vary greatly between countries, meaning that research from one setting can lack generalizability or transferability to other settings. To address this, some studies have examined how integrated care is understood and implemented based on country income, with integrated care seen as a vehicle to improve access to care in lower- and middle-income countries compared with reducing costs in higher-income countries [4]. Integrated care is therefore increasingly a priority within many countries but challenges persist due to the complexity of health and social care systems [5].

Priorities for the future development of integrated care have been reported [6, 7], with a focus on designing and implementing models or approaches to integrated care rather than establishing the research needed to underpin them. There is also a limited understanding of the implementation of integrated care across diverse health care systems and geographical countries. However, shared learning has been demonstrated to be both possible and valuable [4]. It is less clear if the research priorities would help us to better understand how integration of care (cross-boundary working) can be implemented and improved upon. This study aimed to address this evidence gap by developing a consensus on international research priorities related to integration of care and cross-boundary working.

## Methods

### Study design

**Figure 1:**
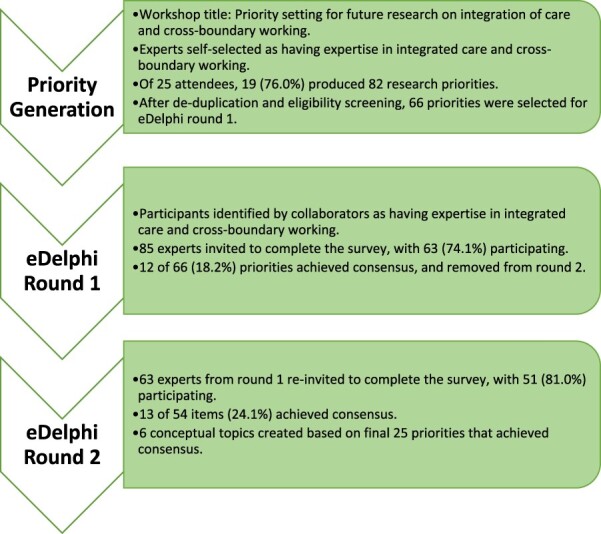
Delphi study process flowchart.

A Delphi study embeds a structured approach to developing consensus amongst subject experts to integrate knowledge, typically consisting of initial priority generation and followed by two or three rounds of consensus development. Within this study, a sequential electronic Delphi (eDelphi) study was conducted, consisting of initial priority generation followed by two rounds of consensus development via an online survey. eDelphi studies are well-established alternatives to traditional Delphi studies within health services research including studies related to patient safety and health care quality. Conducting the study online helped to integrate stakeholder voices across a broad range of continents and countries, and represented different health and social care systems [8]. This study is reported following the Recommendations for the Conducting and Reporting of DElphi Studies (CREDES) guideline [9], which while developed for palliative care is transferable to other settings. Figure 1 shows the full eDelphi process. Ethical approval for the study was provided via Northumbria University’s ethics system (project number: 4693, reference: Scott 2023–4693-3687).

### Participants, recruitment, and sampling

Participants involved in priority generation were delegates of a 45-min workshop at an international conference, the International Society for Quality in Health Care (ISQua) 2023 in Seoul, South Korea. The workshop was titled, ‘Priority setting for future research on integration of care and cross-boundary working’. Conference delegates self-registered to attend the workshop, having been informed in the conference programme that the workshop was for people with expertise in integrated care and cross-boundary working in relation to health care quality and patient safety and that delegates would be discussing and generating priorities for a Delphi study. Delegates were given the option to contribute to the study as a participant or to become a collaborator on the study. Collaborators, consisting of all authors who have expertise in a broad range of research and practise topics associated with health and social care systems, supported the recruitment of relevant participants by identifying and approaching people from their established networks to the first eDelphi round. When identifying potential participants, collaborators were asked to use the following criteria, which were then used by JS to screen potential participants:

1. Expertise in any form of integrated care or cross-boundary working;
2. Relevant background, such as researchers, clinicians, management/leadership roles, and;
3. Have experience with any setting that is relevant to health or social care, such as primary, acute, secondary, emergency, community, long-term residential, mental health or any others deemed relevant to integrated care or cross-boundary working.

### Data collection

#### Priority generation

Priorities were generated during the workshop, facilitated by J.S. and S.W. Attendees were provided with a brief introduction to the workshop objective, and provided with a definition of integrated care and cross-boundary working. The definition was based on the World Health Organization definition of integrated care, adapted to also include social care:

Health [*and social care*] services that are managed and delivered in a way that ensures people receive a continuum of health promotion, disease prevention, diagnosis, treatment, disease management, rehabilitation and palliative care services, at the different levels and sites of care within the health [*and social care*] system, and according to their needs, throughout their whole life. [10]

Attendees were also provided an additional prompt to guide their thoughts and discussion: ‘What are the research priorities in your country and health/social care setting when working together across the different levels and sites of care?’ Attendees then worked in small groups of up to eight people to discuss priorities. Towards the workshop end, attendees were asked to individually complete a brief survey using the JISC Online Survey platform to provide their priorities along with a brief context statement for each priority. Priorities eligible for inclusion in the eDelphi survey had to be relevant to integrated care or cross-boundary working. Eligibility was therefore defined as any priority that could be applied to the aforementioned adapted WHO definition of integrated care [10], with screening conducted by J.S.

#### eDelphi survey

The eDelphi survey was distributed online [11] using the JISC Online Survey platform. A survey link was sent directly to potential participants’ email addresses with an invitation to participate in the study. Participants were then sent two follow-up reminder emails a week apart. Surveys remained open for a total of 3 weeks during each round. In Round 1 of the eDelphi process (October 2023), participants were asked to provide information about the setting(s) they worked in (academia, health care clinician, health care management, or leadership) and the length of time they had worked in the setting(s) as an indicator of expertise. Participants then responded to a seven-point Likert scale (strongly disagree = 1, to strongly agree = 7) for all priorities. In Round 2 (November—December 2023), priorities that had achieved consensus were removed from the survey and reported to participants. The order of priorities presented to participants was randomized. Then, item-by-item consensus scores and graphs showing ratings from Round 1 were presented and participants completed the same seven-point Likert scale on remaining items.

### Data analysis

Initial priorities generated in the workshop were collated and duplicates or priorities with no relevance to integrated care or cross-boundary working were removed. Where priorities were similar, the brief context statements were examined to determine whether they could be combined to ensure novelty. Following removal or combination of duplicates, priorities were then screened for eligibility. The study aimed to achieve consensus rather than stability between rounds, with consensus defined and communicated to participants in each round *a priori* as ≥80% agreement (strongly agree or agree), a score deemed acceptable for achieving consensus in Delphi studies [12]. Participants were informed in advance of the consensus threshold. Upon completion of Round 2, the priorities that reached consensus were conceptually grouped by J.S. and reviewed by all authors to ensure that robust and meaningful recommendations could be made for practice, policy, and research.

## Results

### Priority generation

A total of 25 people attended the workshop, with 19 participants (76%) completing the survey and producing 82 research priorities. Workshop participants represented a diverse sample, with those completing the survey representing 10 countries (Australia, Canada, Ghana, Ireland, Jordan, Nigeria, Norway, Oman, Singapore, and the United Kingdom) across 5 continents. Most workshop participants were researchers/academics (*n* = 12) followed by health care managers (*n* = 4). One participant was a clinician, and another participant reported they held a senior strategic role in their health care system. Following the removal of duplicate topics and a review of eligibility, 66 research priorities were included in the first eDelphi round (16 were excluded or combined). Workshop participants obtained permission from and provided contact details for other experts to take part in the Delphi rounds, resulting in 85 potential respondents.

### Delphi surveys

In Round 1, 63 of 85 (74.1%) experts completed the survey, representing 10 countries (Australia, Canada, Ghana, Ireland, Italy, Jordan, Nigeria, Norway, Papua New Guinea, and the United Kingdom) across 4 continents, with 37 (58.7%) male and 26 (41.3%) female respondents. Experts who completed Round 1 were academics (*n* = 49), health care clinicians (*n* = 25), and/or health care management or leadership (*n* = 29), with a total combined 949 years of experience (range = 1–45, mean = 15.1, standard deviation = 10.2). In Round 1, a total of 12/66 priorities (18.2%) achieved consensus as being priorities, with the remaining 54 priorities presented to participants again in Round 2. Of the 63 experts invited to complete the Round 2 survey, 51 (81.0%) responded, representing the same countries as Round 1 except for Jordan which was had no respondents. The gender distribution of participants remained almost identical, with 30 (58.8%) male and 21 (41.2%) female respondents. In Round 2, a further 13/54 priorities (24.1%) achieved consensus. The final number of priorities that achieved consensus as being priorities for integrated care and cross-boundary working was 25/66 (37.9%). Table 1 provides a country-by-country comparison of the number of people invited, number of respondents, and response rates across the different stages of the study.

**Table 1.** Participant information per stage of study

		Round 1			Round 2^a^	
Country	Workshop (between-country %)	Invited	Responses (between-country %)	Response rate (%)	Responses (between-country %)	Response rate (%)
Australia	1 (5)	9	8 (13)	88.9	7 (14)	87.5
Canada	2 (11)	4	2 (3)	50.0	2 (4)	100
Ghana	1 (5)	6	5 (8)	83.3	5 (10)	100
Ireland	2 (11)	11	9 (14)	81.8	7 (14)	77.8
Italy	0 (0)	1	1 (2)	100	1 (2)	100
Jordan	2 (11)	2	1 (2)	50.0	0 (0)	0
Nigeria	3 (16)	23	19 (30)	82.6	13 (25)	68.4
Norway	2 (11)	7	3 (5)	42.9	3 (6)	100
Oman	1 (5)	1	0 (0)	0	0 (0)	
Singapore	1 (5)	1	0 (0)	0	0 (0)	
United Kingdom	4 (21)	19	14 (22)	73.7	12 (24)	85.7
Papa New Guinea	0 (0)	1	1 (2)	100	1 (2)	100
Total	19	85	63	74.1	51	81.0

a Number of participants invited is the same as the number that responded in round 1.

### Conceptual topics

Priorities that achieved consensus were placed into one of six conceptual topics: (i) access to care, (ii) data sharing and technology, (iii) measurement of care quality, (iv) patient experience and satisfaction, (v) service design, integration, and governance, and (vi) teamwork and leadership (Table 2).

**Table 2.** Research priorities and levels of consensus achieved

	Round 1		Round 2	
Conceptual topics and individual research priorities	Mean (SD)	Consensus %	Mean (SD)	Consensus %
**Access to care (*n* = 5)**				
Encouraging access to primary care as a first point of contact	6.0 (1.4)	73.0	6.2 (1.3)	80.4
Providing equitable access to healthcare for disadvantaged groups	6.5 (1.2)	87.3	N/A	N/A
Providing equitable access to healthcare regardless of patient demographics	6.5 (1.1)	90.5	N/A	N/A
Providing universal health coverage in line with the Sustainable Development Goals	6.3 (1.2)	85.7	N/A	N/A
Strengthening primary healthcare to facilitate universal healthcare coverage	6.3 (1.2)	85.7	N/A	N/A
**Data sharing and technology (*n* = 3)**				
Examine how to integrate data systems to enable integrated care	6.1 (1.3)	81.0	N/A	N/A
Improve data sharing and data linkage across organizational boundaries	6.4 (1.0)	85.7	N/A	N/A
Technology-enabled integrated care to assist in healthcare delivery across different settings of care	6.0 (1.2)	73.0	6.3 (1.0)	84.3
**Measurement of care quality (*n* = 4)**				
Develop quality indicators that operate across settings	6.2 (1.0)	84.1	N/A	N/A
Focus on aligning priorities and gearing operations, performance, and outcomes toward shared goals rather than discipline- or profession-specific goals	5.9 (1.5)	74.6	6.2 (1.2)	84.3
Measurement of longer-term patient outcomes	6.1 (1.1)	76.2	6.4 (1.0)	94.1
Understand the local contextual factors that determine the effectiveness of outcomes	6.2 (1.0)	85.7	N/A	N/A
**Patient experience and satisfaction (*n* = 3)**				
Examine patient safety and satisfaction in integrated care	6.1 (1.2)	82.5	N/A	N/A
How best to involve patients and carers in providing feedback on services when their care crosses organizational boundaries	6.3 (0.9)	85.7	N/A	N/A
Understand patient experience of the current level of service integration	6.1 (1.3)	79.4	6.4 (0.8)	86.3
**Service design, integration and governance (*n* = 7)**				
To better integrate mental health, social care and physical health services	6.3 (1.0)	82.5	N/A	N/A
Co-producing approaches to integrated care with staff and service users	6.1 (1.4)	77.8	6.3 (1.0)	86.3
Cross-contextual studies to understand the system as a whole instead of singular settings	6.1 (1.0)	73.0	6.3 (1.0)	82.4
How/whether local systems with more extensive co-production approaches achieve better outcomes	5.7 (1.4)	68.3	6.1 (1.0)	80.4
Identify incentives to deliver quality care across boundaries	5.9 (1.3)	71.4	6.2 (1.1)	84.3
Inclusion of vulnerable and disabled people in healthcare provision	6.2 (1.2)	76.2	6.5 (0.9)	84.3
Understanding what improvement/improvement capabilities can enable better cross-boundary working and integration	5.7 (1.4)	65.1	5.8 (1.2)	80.4
**Teamwork and leadership (*n* = 3)**				
Develop shared leadership models to overcome cultural and hierarchal barriers to communication among healthcare professionals that impact shared care	6.0 (1.3)	74.6	6.2 (1.1)	84.3
How to train integrated health care teams to operate and share leadership roles	6.2 (1.0)	81.0	N/A	N/A
Team-based training at the undergraduate level to improve teams and professions working together in practice	5.8 (1.5)	66.7	6.3 (1.0)	82.4

N/A = not applicable. SD = standard deviation. Priorities are colour-coded based on final consensus level.

#### Access to care (n = 5 priorities)

Experts indicated that research on ‘providing equity of access to care’ was a priority, whether this was specifically ‘for disadvantaged groups’ (87.3%) or ‘regardless of patient demographics’ (90.5%). Further research priorities were around ‘encouraging access to primary care as a first point of contact’ (80.4%) and ‘strengthening primary healthcare to facilitate universal healthcare coverage’ (85.7%). A final research priority related to access to care was ‘providing universal health coverage in line with the Sustainable Development Goals’ (85.7%).

#### Data sharing and technology (n = 3 priorities)

Priorities were identified by experts as research priorities related to data sharing and technology. There was an identified need to understand how to ‘improve data sharing and data linkage across organisational boundaries’ (85.7%) and ‘examine how to integrate data systems to enable integrated care’ (81.0%). The final priority was for research on ‘technology-enabled integrated care to assist in healthcare delivery across different settings of care’ (84.3%).

#### Measurement of care quality (n = 4 priorities)

Priorities identified for the measurement of care quality included addressing gaps in quality indicators that capture issues across organizational and professional boundaries, including the need to ‘develop quality indicators that operate across settings’ (84.1%) and ‘to focus on aligning priorities and gearing operations, performance, and outcomes toward shared goals rather than discipline- or profession-specific goals’ (84.3%). There was also consensus on the need for research that will ‘measure longer-term patient outcomes’ (94.1%), as well as to ‘understand the local contextual factors that determine the effectiveness of outcomes’ (85.7%).

#### Patient experience and satisfaction (n = 3 priorities)

Research on how to capture patient experience and satisfaction in integrated care and as patients crossed organizational boundaries was recognized by experts to be a priority. Three priorities were identified related to this; ‘examine patient safety and satisfaction in integrated care’ (82.5%), ‘how best to involve patients and carers in providing feedback on services when their care crosses organisational boundaries’ (85.7%), and ‘understand patient experience of the current level of service integration’ (86.3%).

#### Service design, integration, and governance (n = 7 priorities)

Priorities related to service design centred around involvement of patients and staff. These priorities comprised ‘co-producing approaches to integrated care with staff and service users’ (86.3%), and ‘inclusion of vulnerable and disabled people in healthcare provision’ (84.3%). Where co-production is used in service design, an additional priority is to examine ‘how/whether local systems with more extensive co-production approaches achieve better outcomes’ (80.4%). More broadly, experts agreed on the need for research to ‘better integrate mental health, social care and physical health services’ (82.5%), to ‘understand what improvement/improvement capabilities can enable better cross-boundary working and integration’ (80.4%) and ‘for cross-contextual studies to understand the system as a whole instead of singular settings’ (82.4%). Finally, there was consensus on the need to ‘identify incentives to deliver quality care across boundaries’ (84.3%).

#### Teamwork and leadership (n = 3 priorities)

The need for research related to teamwork and leadership around integrated care and cross-boundary working was identified. Two priorities related to training both pre-registration and post-registration, including further research on ‘team-based training at the undergraduate level to improve teams and professions working together in practice’ (82.4%), and ‘how to train integrated health care teams to operate and share leadership roles’ (81.0%). Separately from training, there was consensus on an identified need to ‘develop shared leadership models to overcome cultural and hierarchal barriers to communication among healthcare professionals that impact shared care’ (84.3%).

## Discussion

### Statement of principal findings

**Figure 2:**
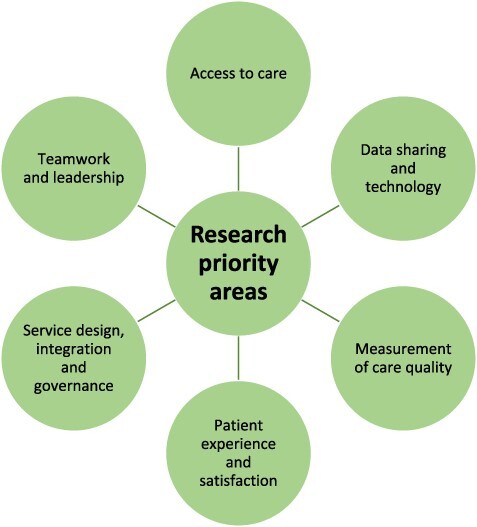
Six conceptual topics that are research priorities for integrated care and cross-boundary working.

This study has developed consensus on 25 international research priorities related to integration of care and cross-boundary working. These priorities span six conceptual topics, presented in Fig. 2. In the following section, we examine a selection of current advances relevant to each of these conceptual topics, though we also encourage readers to consider each individual priority.

### Interpretation within the context of wider literature

The first conceptual topic, ‘access to care’, included providing equitable access to health care. This is a persistent problem relevant to many, if not all, countries and health care systems and is a key sustainable development goal. Individual, structural, and systematic factors contribute to poor access to services [13], though evidence suggests integrated models of care can contribute to improved access [14]. Recently, emphasis has been placed on improving equity in integrated care that incorporates implementation science [15]. The second topic, ‘data sharing and technology’, highlights the need for research to address the existing practical, organizational, and regulatory challenges that are a known barrier to successful integrated care [16]. Our study identified that this needs to encompass both the underlying organizational data systems while also developing new approaches to technology-enabled integrated care, which could also help to address equitable access to care and climate sustainability via telehealth [17].

The third topic, ‘measurement of care quality’, highlights the importance of research on the collection of longitudinal patient outcome data. For example, the utilization of measurement-based care for mental health conditions can improve communication between providers and patients, facilitate patient involvement, and inform quality improvement [18]. Further, collaboration processes are best measured over time [19], and clinical practice guidelines, from which quality indicators can be derived, can enable cross-boundary collaboration [20]. Linked to measuring care quality was the fourth topic, ‘patient experience and satisfaction’. This highlights the importance of capturing data on how patients perceive integrated care services; a recent review of patient experience of integrated care in the United Kingdom highlighted a lack of person-centred care coordination when considered from patients’ perspectives [21].

Patient experience extends into the fifth topic of ‘service design, integration and governance’, where co-designing services with patients and professionals were deemed to be a research priority. A value-based approach includes integrated services being collaborative and co-produced, with shared responsibility and accountability [5, 22]. Further research is required on how this can be best achieved, as involving patients is also important for producing resilient organizations [23]. Further, greater investment by government and industry is needed to support workforce development in primary and community care settings, so that the focus is on health management, disease prevention, and patient quality of life, especially for chronic and complex conditions [24]. Governance structures and policy frameworks need to support rather than restrict cross-boundary collaboration and, linked to the earlier 'data sharing and technology’ topic, support interoperable technology for a ‘whole-system’ approach [25].

The final topic, ‘teamwork and leadership’, highlights the need for developing shared values across multiple professional groups rather than discipline-specific goals, which should be a core component of training curricula [26]. In most countries, education in medicine, nursing, social care, and allied health are delivered separately which can contribute to communication barriers, siloed practices, and lack of knowledge or role ambiguity; correcting this has been recognized to be a building block of integrated care [27]. Integrated care systems have also been developed with little consideration of the leadership approaches required for their delivery [28]. Given that leadership is considered to be central to the functioning of integrated health care systems [29], it should be a focus for further research.

### Implications for policy, practice, and research

Our findings have implications that span macro (policy), meso (behavioural and cultural), and micro (individual practice) levels of health and social care systems, which are important for the successful integration of care [28]. The priorities can be used by researchers to inform the direction of future research on integrated care and cross-boundary working, regardless of country and health system. Health systems are generally adapting to changes in population health, with increased focus on chronic conditions and associated multimorbidities that lead to increased service utilization [30]. While there is evidence of improved patient outcomes at a micro level as a result of integrating care in high-income countries, there is still a stark dearth of evidence in lower- and middle-income countries [31]. Policymakers and practitioners, as potential leaders for integrated care, could therefore consider our identified priorities when planning, designing, and delivering services, and incorporate research into these activities to ensure that they are fully understood. Successful development of integrated services is highly context-specific [16] with implementation likely to encounter significant challenges in lower- and middle-income countries [31] and therefore policymakers and practitioners will need to consider these research priorities in the context of their setting, whether this spans national, regional, or local systems.

### Strengths and limitations

A key strength of this eDelphi study is that we were able to recruit participants from multiple countries across five continents, including a relatively high level of representation from lower- and middle-income countries. Another key strength is that we obtained a high response rate across all stages of the eDelphi process. A 70% response rate is required for robust Delphi studies [32], which was achieved at each stage despite international Delphi studies having higher levels of attrition [8]. This response rate may have been facilitated by working closely with in-country collaborators to recruit experts across various countries, which can be utilized by others conducting similar international eDelphi studies in the future.

A limitation is that the initial priorities were generated at a research conference, albeit one focused on the quality and safety of health care with an international audience. The initial priorities generated are therefore unlikely to be exhaustive of all research priorities related to integrated care and cross-boundary working, particularly as not all countries were represented. Furthermore, the nature of the conference meant that delegates were more likely to represent health care than social care, meaning that priorities may not fully consider priorities for integration of care and cross-boundary working from other perspectives. While some participants did have expertise in non-health care settings such as social care, criminal justice systems, and community-based mental health, these perspectives remain under-represented. Another limitation was that patients and service users were not represented in the study. It is imperative that voices within health and social care sectors, including patients, service users, and carers or family members, are equally represented in priority-setting exercises. Future research should look to specifically examine research priorities among the different groups, including where differences exist. Further, there was consensus on involving patients and service users in the co-design of services, and this should also be extended to patient and service user involvement in research on integrated care and cross-boundary working.

## Conclusions

We have developed a consensus on 25 future research priorities for integrated care and cross-boundary working from experts representing a range of geographical regions and health and social care systems. From these priorities, six broad topics were derived that should be considered by researchers when designing and conducting studies, and policymakers and practitioners when planning, designing, and delivering services. Integrating care services and improving cross-boundary working support enhanced care provision regardless of country, and the topics and priorities identified in this study can help inform policymakers, practitioners, and researchers across the world in the pursuit of improved integrated care systems.

## Data Availability

The data underlying this article will be shared upon reasonable request to the corresponding author.
